# Non-Vitamin K Antagonist Oral Anticoagulants versus Low Molecular Weight Heparin for Cancer-Related Venous Thromboembolic Events: Individual Patient Data Meta-Analysis

**DOI:** 10.3390/cancers15245887

**Published:** 2023-12-18

**Authors:** Chun En Yau, Chen Ee Low, Natasha Yixuan Ong, Sounak Rana, Lucas Jun Rong Chew, Sara Moiz Tyebally, Ping Chai, Tiong-Cheng Yeo, Mark Y. Chan, Matilda Xinwei Lee, Li-Ling Tan, Chieh-Yang Koo, Ainsley Ryan Yan Bin Lee, Ching-Hui Sia

**Affiliations:** 1 Yong Loo Lin School of Medicine, National University of Singapore, Singapore 119228, Singapore; e0886363@u.nus.edu (C.E.Y.); cheneelow@u.nus.edu (C.E.L.); sounak_rana@u.nus.edu (S.R.); lucasc@u.nus.edu (L.J.R.C.); ping_chai@nuhs.edu.sg (P.C.); tiong_cheng_yeo@nuhs.edu.sg (T.-C.Y.); mark.chan@nus.edu.sg (M.Y.C.); ainsleyryanlee@u.nus.edu (A.R.Y.B.L.); 2 Division of Cardiology, Department of Medicine, Ng Teng Fong General Hospital, Singapore 609606, Singapore; 3 Department of Cardiology, National University Heart Centre, Singapore 119074, Singapore; li_ling_tan@nuhs.edu.sg (L.-L.T.); christopher_koo@nuhs.edu.sg (C.-Y.K.); 4 Department of Haematology-Oncology, National University Cancer Institute, Singapore 119228, Singapore; matilda_lee@nuhs.edu.sg

**Keywords:** anticoagulation, NOAC, LMWH, Meta-analysis

## Abstract

**Simple Summary:**

Low molecular weight heparin (LMWH) has been the standard of care for venous thromboembolism (VTE) but new guidelines approved using non-vitamin K antagonist oral anticoagulants (NOAC). By conducting an individual patient data meta-analysis of randomised controlled trials (RCTs) comparing the outcomes of NOAC versus LMWH in cancer patients, we aim to determine an ideal strategy for the prophylaxis of VTE and prevention of VTE recurrence. Our study further addresses the conflicting evidence in the literature with an individual patient data meta-analysis. However, other studies are required to balance the risk of recurrent VTE and bleeding among different cancer subgroups. Emerging data highlight the need for individualised antithrombotic strategies to achieve optimal management of cancer patients.

**Abstract:**

Venous thromboembolism (VTE) is a leading cause of morbidity and mortality in cancer patients. Low molecular weight heparin (LMWH) has been the standard of care but new guidelines have approved the use of non-vitamin K antagonist oral anticoagulants (NOAC). By conducting an individual patient data (IPD) meta-analysis of randomised controlled trials (RCTs) comparing the outcomes of NOAC versus LMWH in cancer patients, we aim to determine an ideal strategy for the prophylaxis of VTE and prevention of VTE recurrence. Three databases were searched from inception until 19 October 2022. IPD was reconstructed from Kaplan–Meier curves. Shared frailty, stratified Cox and Royston–Parmar models were fit to compare the outcomes of venous thromboembolism recurrence and major bleeding. For studies without Kaplan–Meier curves, aggregate data meta-analysis was conducted using random-effects models. Eleven RCTs involving 4844 patients were included. Aggregate data meta-analysis showed that administering NOACs led to a significantly lower risk of recurrent VTE (RR = 0.65; 95%CI: 0.50–0.84) and deep vein thrombosis (DVT) (RR = 0.60; 95%CI: 0.40–0.90). In the IPD meta-analysis, NOAC when compared with LMWH has an HR of 0.65 (95%CI: 0.49–0.86) for VTE recurrence. Stratified Cox and Royston–Parmar models demonstrated similar results. In reducing risks of recurrent VTE and DVT among cancer patients, NOACs are superior to LMWHs without increased major bleeding.

## 1. Introduction

Venous thromboembolism (VTE) is one of the leading causes of mortality in patients with cancer [1]. Cancer patients have a higher risk of cancer-associated VTE compared to the general population [2]. Patients who develop VTE during the cancer diagnosis tend to have much worse outcomes as compared to those without VTE [3]. As the risk of recurrent VTE in the initial months after cancer diagnosis is high, there is a need for long-term treatment and prophylaxis with anticoagulation. Acute treatment and prophylaxis with low molecular weight heparin (LMWH) has been the standard of care [4,5] for cancer patients at risk of VTE. However, there have been challenges with patient adherence to long-term subcutaneous administration of LMWH [6]. Moreover, it is uncertain if LMWH therapy has a benefit beyond 6 months. Furthermore, LMWH has its set of inconveniences and limitations, namely the inability to use it in patients with severe renal impairment and thrombocytopenia [7]. These, coupled with the inconvenience of daily subcutaneous injections, makes LMWH a less appealing option [8,9].

Non-vitamin K antagonist oral anticoagulants (NOACs), such as apixaban, dabigatran, edoxaban and rivaroxaban, have been used for acute treatment of VTE in the general population. These medications were preferred over warfarin due to an association with a favourable safety profile and efficacy [10]. NOACs in clinical use today include direct thrombin inhibitors and direct factor Xa inhibitors [7,11]. To date, NOACs have demonstrated comparable or superior outcomes to the conventional vitamin K antagonist, warfarin, in certain populations, such as the prevention of stroke in patients with non-valvular atrial fibrillation, and have been increasingly used in various clinical settings [12,13].

However, less is known about the efficacy and safety of NOACs in cancer patients. As cancer patients have a higher risk of anticoagulant-related bleeding complications [14], recent trials [15,16] have explored the efficacy and safety of NOACs for treatment of VTE in cancer patients. New guidelines [17,18,19] have approved the use of certain NOACs for treatment of VTE in cancer patients. There remains conflicting evidence [20] of the safety and efficacy profiles reported by different trials, and the superiority of NOACs over LMWHs remains inconclusive. Although those trials have reported conflicting results, it could be potentially due to differing patient selection criteria.

With the release of data from new trials, we performed a one-stage and aggregate data meta-analysis of existing randomised controlled trials. Although there were prior meta-analyses conducted, no meta-analysis to date has used individual patient data in the analysis of the trials published. The results of a meta-analysis of aggregate data can be less granular and limited by the lack of individual patient data [21]. As such, we attempt to conduct a pooled analysis using reconstructed individual patient data from the available RCTs to obtain accurate estimates of the safety and efficacy outcomes of NOACs and LMWHs for VTE treatment in cancer patients. By aggregating the data from available clinical trials using an individual patient data meta-analysis, we aim to determine an ideal strategy for the prophylaxis of VTE and prevention of VTE recurrence in cancer patients.

## 2. Methods

### 2.1. Search Strategy

The Preferred Reporting Items for Systematic Reviews and Meta-Analyses (PRISMA) guidelines [22] informed the design and execution of this study. The protocol was registered on PROSPERO (CRD42022381937). MEDLINE, Embase and Cochrane Central Register of Controlled Trials (CENTRAL) were searched from inception until 19 October 2022. Keywords related to cancer, thrombosis, embolism, venous occlusion, non-vitamin K antagonist oral anticoagulants and low molecular weight heparin were used for the search. The full EMBASE search strategy is reported in Supplementary Data S1. References, from included studies and review papers, were systematically hand searched to include studies omitted by the electronic search.

### 2.2. Eligibility Criteria

**Figure 1:**
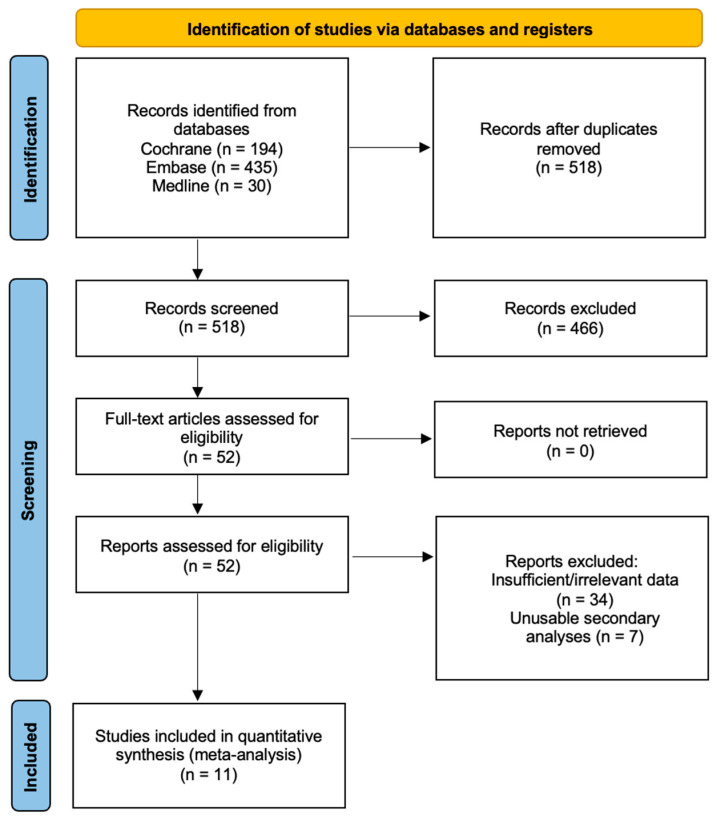
Preferred Reporting Items for Systematic Reviews and Meta-Analyses (PRISMA) flowchart.

We included all English language peer-reviewed randomised-controlled trials comparing the outcomes of NOAC versus LMWH in cancer patients. Studies were excluded if they were (a) non-RCTs or studies that did not provide sufficient data for the control group or (b) case reports, case series, reviews, observational studies or letters. Secondary analyses of included RCTs were screened for usable data. The selection process is shown in Figure 1.

### 2.3. Data Extraction

The titles and abstracts of potential articles were screened against the eligibility criteria independently by two authors, and any discrepancies were resolved by discussion with a third author. The full texts of all potentially eligible studies were then retrieved and reviewed by two independent reviewers, with any discrepancies resolved by common consensus.

Baseline information including age, gender and comorbidities were collected. Extracted outcomes included incidence of death, death related to pulmonary embolism, death related to bleeding, death related to venous thromboembolism, major bleeding, clinically non-major relevant bleeding, deep vein thrombosis, non-fatal pulmonary embolism, fatal pulmonary embolism, recurrent venous thromboembolic events, recurrent deep vein thrombosis, venous thromboembolism, and recurrent pulmonary embolism. We defined major bleeding as acute clinically overt bleeding associated with ≥ one of the following: a decrease in the haemoglobin level of at least 2 g per decilitre; transfusing ≥two units of red cells; bleeding at a critical site (intracranial, intraspinal, intraocular, pericardial, intraarticular, intramuscular with compartment syndrome or retroperitoneal); bleeding resulting in surgical intervention; or fatal bleeding, all occurring during the trial-drug period to 72 h after the last dose was administered [23], while clinically relevant non-major bleeding was defined as overt bleeding that did not meet the criteria for major bleeding but was associated with medical intervention, unscheduled contact with a physician, interruption or discontinuation of study drug, or discomfort or impairment of activities of daily living [24].

### 2.4. Statistical Analysis

The individual patient data (IPD) from published Kaplan–Meier curves was reconstructed using Guyot et al.’s [25] graphical reconstruction method before performing the one-stage meta-analyses. We used digitised images of Kaplan–Meier curves and determined step functions and timing values. To retrieve survival data for individual patients, we used the numerical solutions for the inverted Kaplan–Meier product-limit equations. The reconstructed individual patient data (IPD) set was visually compared to the original curves for verification (Supplementary Data S2) and compared to the original log-rank values.

We quantitatively pooled and analysed the results using RStudio according to the general approaches laid out in the Cochrane Handbook [26]. We performed a two-stage meta-analysis, conducted with the R package metabin (version 5.2-0), and evaluated between-study heterogeneity using I^2^ and τ^2^ statistics. Nominal statistical significance was indicated by two-sided *p* values of <0.05.

We calculated cumulative incidence and overall survival using the Kaplan–Meier method. We employed stratification and shared frailty approaches to account for heterogeneity between studies. We based the main analysis for cumulative incidence on the shared frailty Cox model, which assumes similar risks of failure for patients within the same study [27]. We conducted a secondary analysis using the stratified Cox model, which models heterogeneity between studies by estimating a baseline hazard for patients from each included study. We assessed the validity of the proportional hazards assumption using the Grambsch–Therneau test for a non-zero slope [28] and by plotting scaled Schoenfield residuals [29]. As a sensitivity analysis, we analysed the pooled data using the parametric Royston–Parmar [30] models and conducted restricted mean survival time (RMST) analysis.

We also performed aggregate data meta-analysis for outcomes which were not captured and reported in the form of Kaplan–Meier curves, as IPD reconstruction necessary for one-stage meta-analysis was not possible for these data. Subgroup analysis by cancer type was not conducted due to heterogeneity of the reported outcomes.

We also conducted an exploratory calculation of the number needed to treat (NNT) from the results of the one-stage and two-stage meta-analysis for the main outcomes of recurrent venous thromboembolism, recurrent deep vein thrombosis and major bleeding. We used the “nnt” R package to calculate the NNT with the reconstructed IPD data from the one-stage meta-analysis, with 180 days as the clinical timepoint of interest as that is the approximate mean duration of follow-up. We used the “meta” package to calculate NNT from the results of the two-stage meta-analysis. For this analysis, we used the control group event probabilities from CARAVAGGIO study [23] as it has the largest number of patients included in the control arm. The calculated NNT values are presented as number needed to treat for an additional beneficial outcome (NNTB), and number needed to treat for an additional harmful outcome (NNTH). Confidence intervals of 95% are reported. As outlined by Altman et al. [31], if the confidence intervals of the risk ratio or the hazard ratio include the null effect of the summary measure (risk ratio = 1 or hazard ratio = 1), we present the NNT confidence interval in the format of estimated NNT (lower bound of NNTB to ∞; lower bound of NNTH to ∞).

### 2.5. Risk of Bias Assessment

Risk of bias in the studies was assessed independently by two reviewers using the Cochrane tool for assessing risk of bias in randomised trials (RoB 2, 2019) [32], which includes the appraisal for inclusion criteria, baseline characteristics reporting, relevant outcome reporting and measurement of the condition and appropriateness of statistical analyses. Discrepancies were resolved by discussion with a third author. Although there was some bias, most of the studies were judged to be at low risk of bias (Supplementary Figure S1).

## 3. Results

The PRISMA flow diagram is presented in Figure 1. The search strategy identified 518 studies after removal of duplicates, of which 52 were included in the full-text review. A total of 41 studies were excluded, with 34 out of the 41 excluded due to insufficient data and the remaining 7 unusable for secondary analyses. Ultimately, we included data from 11 individual studies [7,15,16,23,24,33,34,35,36,37,38].

### 3.1. Baseline Characteristics

All included studies were published between 2015 and 2022. The participant baseline characteristics are shown in Table 1. Across the 11 individual studies, a total of 4844 patients were included. Of these, 2456 patients were randomised to NOAC, while 2388 patients were randomised to LMWH. Of the eleven studies, there were five international trials, two from USA and one each from China, Europe, Egypt and France (Table 1). The type of cancers studied includes prostate, gastrointestinal, skin, respiratory, gynaecological, haematological, urogenital, breast, head and neck and brain. There were five studies using apixaban, three on rivaroxaban, one study each on betrixaban and edoxaban, and one study used both rivaroxaban and apixaban. Six studies looked at dalteparin, four on enoxaparin and one study on nadroparin.

### 3.2. Aggregate Data Meta-Analysis

**Figure 2:**
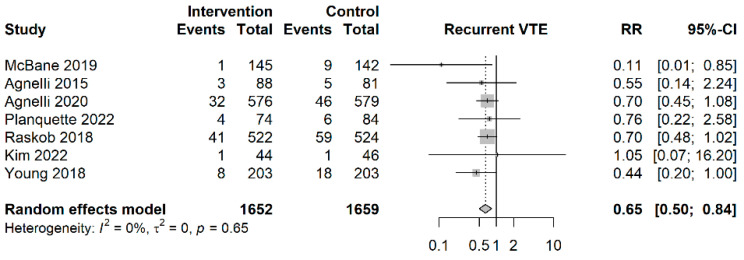
Forest plot of recurrent venous thromboembolism outcomes [7,15,16,23,24,35,37]. Vertical reference line indicates a risk ratio of 1. Diamond represents the aggregated effect size.

As shown in Figure 2, patients in the NOAC group had a significantly lower risk of recurrent venous thromboembolism compared to the patients in the LMWH group (RR = 0.65; 95%CI: 0.50–0.84, I^2^ = 0%). The estimated NNT was NNTB 35.8 (95%CI: NNTB 25.3–NNTB 77.2).

**Figure 3:**
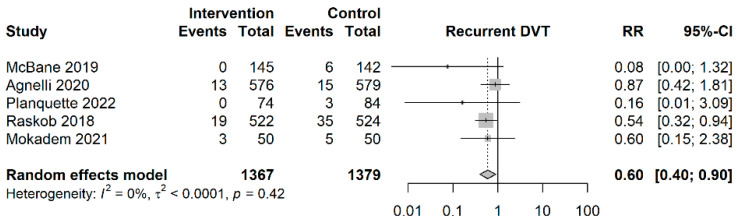
Forest plot of recurrent deep vein thrombosis outcomes [7,15,23,36,37]. Vertical reference line indicates a risk ratio of 1. Diamond represents the aggregated effect size.

As shown in Figure 3, patients in the NOAC group had a significantly lower risk of recurrent deep vein thrombosis compared to the patients in the LMWH group (RR = 0.60; 95%CI: 0.40–0.90, I^2^ = 0%). The estimated NNT was NNTB 95.7 (95%CI: NNTB 64.0–NNTB 377.6).

**Figure 4:**
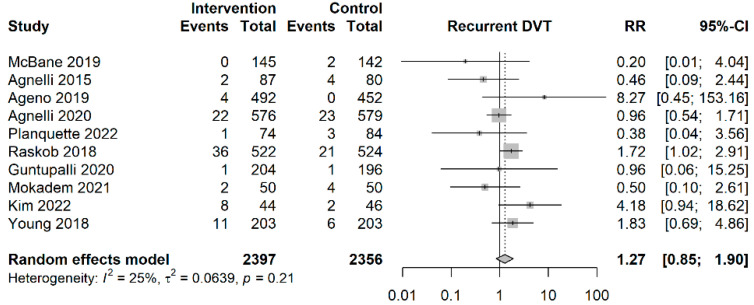
Forest plot of major bleeding outcomes [7,15,16,23,24,33,34,35,36,37]. Vertical reference line indicates a risk ratio of 1. Diamond represents the aggregated effect size.

No significant difference in incidence of death was found between patients in the NOAC group and patients in the LMWH group (RR = 1.03; 95%CI: 0.90–1.18, I^2^ = 0%). The differences in incidences of death related to pulmonary embolism, bleeding and venous thromboembolism between patients in the NOAC group and patients in the LMWH group were insignificant (RR = 1.25; 95%CI: 0.34–4.67, I^2^ = 0%; RR = 0.58; 95%CI: 0.16–2.08, I^2^ = 0%; RR = 1.08; 95%CI: 0.47–2.46, I^2^ = 0%, respectively). No significant difference in incidence of major bleeding or clinically relevant non-major bleeding was found between patients in the NOAC group and patients in the LMWH group (RR = 1.27; 95%CI: 0.85–1.90, I^2^ = 25% (Figure 4); RR = 1.25; 95%CI: 0.89–1.76, I^2^ = 46%, respectively). The estimated NNT for major bleeding was NNTH 94.1 (95%CI: NNTB 163.2–∞; NNTH 28.0–∞). There was no significant difference in incidence of non-fatal or fatal pulmonary embolism between patients in the NOAC group and patients in the LMWH group (RR = 0.39; 95%CI: 0.12–1.24, I^2^ = 0%; RR = 1.25; 95%CI: 0.34–4.67, I^2^ = 0%;). No significant difference in the incidence of venous thromboembolism or recurrent pulmonary embolism was found between patients in the NOAC group and patients in the LMWH group (RR = 0.71; 95%CI: 0.37–1.36, I^2^ = 18%; RR = 0.71; 95%CI: 0.47–1.06, I^2^ = 0%, respectively) (Supplementary Data S3).

### 3.3. One-Stage Meta-Analysis

**Figure 5:**
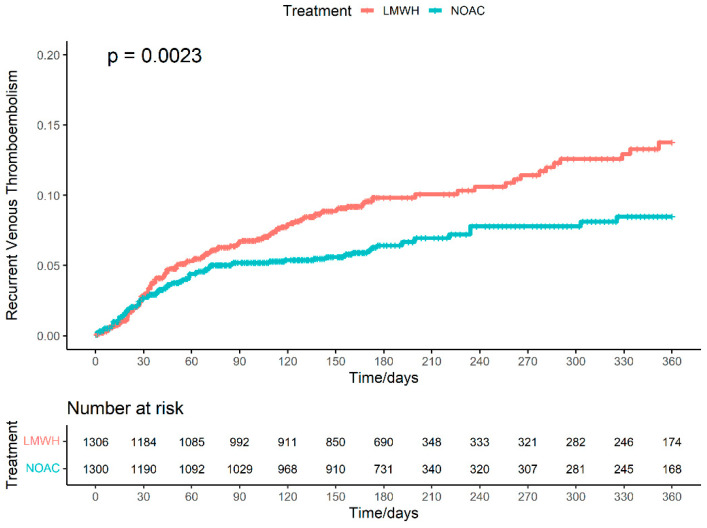
Cumulative incidence curve of pooled cohorts for recurrent venous thromboembolism.

Three included individual studies [15,16,23] published Kaplan–Meier curves for the outcome of venous thromboembolism recurrence. We reconstructed the IPD from the three studies, with a total of 2606 patients and 204 events. Of 1300 patients treated with NOAC, 81 events were recorded during the follow-up period of 238,651 patient-days. Of 1306 patients treated with LMWH, 123 events were recorded during the follow-up period of 234,292 patient-days. Under the shared frailty model (Figure 5), NOAC when compared to LMWH has an HR of 0.65 (95%CI: 0.49–0.86) of recurrent venous thromboembolism. Under the stratified Cox model, NOAC has an HR of 0.65 (95%CI: 0.49–0.86). Under the parametric Royston–Parmar model, NOAC has a significant increase in the RMST (9.94 days; 95%CI: 2.87–17.02). The estimated NNT was NNTB 29.4 (95%CI: NNTB 17.2–NNTB 100).

**Figure 6:**
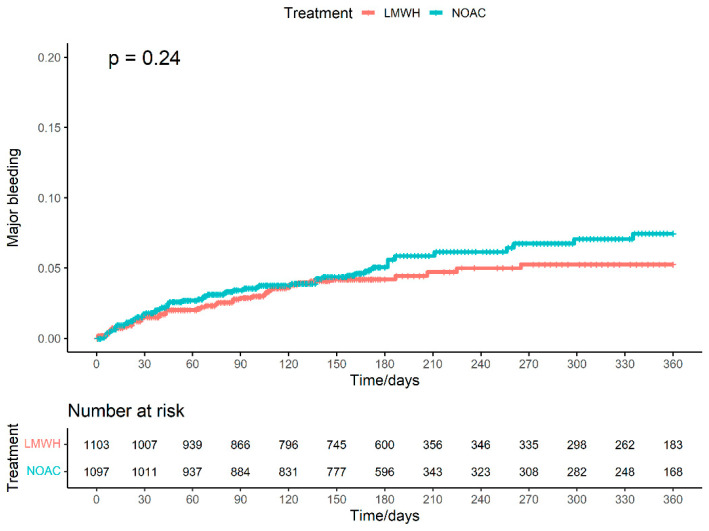
Cumulative incidence curve of pooled cohorts for major bleeding.

Two included individual studies [15,23] published Kaplan–Meier curves for the outcome of major bleeding. We reconstructed the IPD from the two studies, with a total of 2200 patients and 100 events. Of 1097 patients treated with NOAC, 56 events were recorded during the follow-up period of 211282 patient-days. Of 1103 patients treated with LMWH, 44 events were recorded during the follow-up period of 212116 patient-days. Under the shared frailty model (Figure 6), NOAC when compared to LMWH has an HR of 1.27 (95%CI: 0.86–1.88) of major bleeding.

Under the stratified Cox model, NOAC has an HR of 1.27 (95%CI: 0.86–1.89). Under the parametric Royston–Parmar model, NOAC has an insignificant decrease in the RMST (−3.50 days; 95%CI: −9.56–2.56). The estimated NNT was NNTH 111.1 (95%CI: NNTB 90.9–∞; NNTH 34.5–∞).

## 4. Discussion

This systematic review and meta-analysis suggests that NOAC confers a significantly lower risk of recurrent venous thromboembolism and recurrent deep vein thrombosis compared to the LMWH group. Included studies also explored the incidences of major bleeding, clinically relevant non-major bleeding, pulmonary embolism, non-fatal pulmonary embolism, fatal pulmonary embolism, recurrent pulmonary embolism and deep vein thrombosis. We have shown that the incidences of these events were insignificantly different between the two treatment groups. Previous meta-analyses provided contrasting evidence regarding the safety and efficacy of NOACs against LMWHs among cancer patients. In a network meta-analysis, Rossel et al. [39] revealed that NOACs were insignificant in reducing risk of recurrent VTE with no increased risk in major bleeding compared with LMWHs. Fuentes et al. [40] found a lower risk of recurrent VTE when using NOACs compared to LMWHs, while noting an increased risk of major bleeding.

To address the conflicting evidence and guide clinical management, to the best of our knowledge, our study is the first individual patient data meta-analysis to compare the impact of NOACs against LMWHs in cancer patients. A recent meta-analysis of four RCTs by Camilli et al. [41] reported that NOACs were superior to LMWHs in reducing risk of venous thromboembolism with no increase in risk of major bleeding. Their study was limited by a small number of studies, potentially lowering the precision of estimates. Our study included 11 RCTs and reported similar findings.

Interestingly, in a meta-analysis of 11 RCTs that included patients without cancer, Robertson et al. demonstrated a reduced risk of major bleeding with NOACs compared to LMWHs. Cancer patients may have unique predisposing factors, such as previous history of radiation therapy, surgery, metastatic tumours and thrombocytopenia, that predisposes them to a higher risk of major bleeding [42]. Despite advancements in cancer-directed therapy, various large studies have confirmed that bleeding risks are significantly higher in cancer patients [43]. Among a cohort of 3,282,140 cancer patients, Angelini et al. [44] found that cancer patients had higher bleeding incidence than non-cancer patients who are on anticoagulant therapy.

The increased incidence of major bleeding among cancer patients found in previous meta-analyses could also be explained in part by the results of the ADAM-VTE [7] and CARAVAGGIO [23] trials. These two studies included gastrointestinal cancer patients, who are known to have the highest increase in major bleeding [44,45]. Two meta-analyses [46,47] have previously reported a significant association between NOACs and gastrointestinal bleedings in cancer patients. It is particularly important to explore the safety profiles of NOACs in sufficiently powered randomised controlled trials, similar to the PRIORITY trial by Kim and colleagues [35]. In this Phase II trial, which included patients who had advanced upper gastrointestinal tract, hepatobiliary or pancreatic cancer, the primary outcome of clinically relevant bleeding occurred in 34.1% of the patients in the NOAC group, compared to 13.0% of the patients in the group receiving the LMWH, dalteparin. Major bleeding occurred in 18.2% of the patients in the NOAC group and in 4.3% of the patients in the dalteparin group. There was no significant difference in outcomes between the patients treated with rivaroxaban and apixaban. This stands in contrast with some of the existing literature. For example, the CARAVAGGIO [23] study showed that apixaban did not have an increased risk of major bleeding (3.8% of the patients in the apixaban group and 4.0% of the dalteparin group [hazard ratio, 0.82; 95%CI, 0.40 to 1.69]) or clinically relevant non-major bleeding (9.0% of the patients in the apixaban group and 6.0% of the patients in the dalteparin group [hazard ratio, 1.42; 95%CI, 0.88 to 2.30]). However, in the ADAM-VTE [7] trial, apixaban patients had a higher rate of clinically relevant non-major bleeding (6.2% in the apixaban group and 4.2% in the dalteparin group). There still remains much debate about the reasons behind this apparent discrepancy in the risk of major bleeding, clinically relevant bleeding and bruising between these trials.

Future trials should aim to recruit a larger proportion of patients most at risk of these events, such as patients with GI, pancreatic and hepatobiliary cancers. These seemingly contradictory results from the studies should guide future studies to investigate the mechanisms behind the interaction between bleeding and cancer types [48]. Another possible direction would be to explore how the cancer location, instead of the cancer type, could affect bleeding rates after anticoagulation therapy. In the PRIORITY trial [35], cancer involvement at the gastrointestinal mucosa rather than cancer type was a significant risk factor for clinically relevant bleeding (hazard ratio, 2.57). Others have hypothesised that this could be due to the confluence of factors such as high concentrations of NOACs in the gastrointestinal tract, coupled with the damage induced by chemotherapy targeting gastrointestinal tract cancer [35,49,50]. Thromboembolic phenomena are one of many deleterious ways cancer and its treatment can affect the cardiovascular system [51,52]. Overall, cancer and its treatment are associated with a host of adverse effects that clinicians must be cognisant of in order to optimise patient care [53,54]. 

Future research can also delve deeper into understanding how the mode of delivery of the drugs, such as oral administration for NOACs versus parenteral administration for LMWHs, can affect compliance and safety outcomes. An example of this can be found in the ADAM-VTE trial conducted by McBane and colleagues [7]. In this study, investigators incorporated monthly surveys to gauge patient satisfaction with the anticoagulation regimen and the prevalence of bruising. In the initial month, there were emerging reasons for the patients’ preference for NOACs. The parenteral delivery of LMWHs increased the stress and anxiety of patients, leading to a reduction in the patients’ quality of life. As such, patients assigned to LMWHs were three times more likely to terminate their treatment compared to those on NOACs. Given the increasing emphasis on involving patients in the decision making process, it is vital that future trials and healthcare providers consider the psychosocial effects of the medication regimen. This may prove detrimental to intention-to-treat analyses and in real world settings as poor compliance to the administration of medications may result in an inadequate therapeutic effect of anticoagulation. This would also prove to be a valuable area of focus in future clinical trials.

The included studies also often mentioned the problem of statistical underpowering due to a variety of reasons. For example, the CASTA DIVA [37] trial was prematurely terminated due to the slow recruitment rate. This caused it to be unable to reach its predefined criteria of noninferiority. The CARAVAGGIO [23] trial was designed with sufficient statistical power for the primary outcome of recurrent venous thromboembolism and not bleeding events. Thus, it was unable to make definitive conclusions about the risk of bleeding events, which occurred far less commonly. Thus, this meta-analysis aggregating the results of 11 RCTs serves to mitigate the issue of underpowered primary studies.

Our review faced several limitations. We utilised reconstructed rather than primary individual patient records for IPD analysis. The data was not sufficiently granular to perform subgroup analysis of the included patients, and patients of certain demographics may not have benefited from NOACs. Additionally, there was also insufficient data for stratification by cancer type, and we are unable to determine if NOACs are preferentially beneficial or disadvantageous in certain cancer types, such as gastrointestinal cancer. This could have increased the heterogeneity in the results. For example, fewer patients with upper-gastrointestinal malignancy were included in thee ADAM-VTE trial, which, as explained above, could have an increased risk of major bleeding. This possibly led to a lower-than-expected rate of major bleeding in the treatment arms in the ADAM-VTE trial. Moreover, the included studies spanned a range of countries and baseline patient characteristics. Another possible source of heterogeneity could be due to slight variations in the exclusion criteria applied by each trial. The AMPLIFY trial excluded patients with a life expectancy of less than 6 months, the APEX trial excluded patients with intracerebral neoplasms or metastases, or active lung cancer, and the CARAVAGGIO trial excluded patients with brain tumours and acute leukaemia. Thus, the stringency and definition of the inclusion criteria could have influenced the bleeding outcomes and the venous thromboembolism rates in the studies. This could also potentially reduce the generalisability of the results to these groups of patients. It should be noted that a few of the studies considered the long-term administration of placebo injections to be inappropriate, and that they were conducted with an open-label design [7,15]. Nonetheless, they had taken the due measures to mitigate potential bias, such as adjudicating the events while being blinded to the treatment assignments. A possible area of future of research would be to compare the safety and efficacy profiles of direct thrombin inhibitors such as dabigatran against direct factor Xa inhibitors. This could not be performed in the present meta-analysis as all included studies used direct factor Xa inhibitors. Another possible area for subsequent research would be to consider the interactions between NOACs and other drugs, such as antiplatelets or other antithrombotics, that patients are currently prescribed for other comorbidities. Moreover, future trials can consider exploring how hepatic and renal functions affect the safety and efficacy of NOACs. The included studies implemented strict exclusion criteria, mainly exploring the performance of NOACs in patients without renal impairment [24] or hepatic impairment [37]. Upcoming trials can consider investigating how tailoring doses according to renal function [36] can affect the NOAC efficacy.

## 5. Conclusions

In this updated aggregate data meta-analysis and IPD analysis of the safety profile and efficacy of NOACs compared to LMWHs, we found that NOACs are superior to LMWHs in reducing the risk of recurrent VTE and DVT among cancer patients, without an increase in risk of major bleeding. Our study further addresses the conflicting evidence in the literature with an individual patient data meta-analysis. However, other large-scale studies are still required to understand how to balance the risk of recurrent VTE and bleeding among the different cancer subgroups. Emerging data highlight the need for individualised antithrombotic strategies to achieve optimal management of cancer patients.

## Figures and Tables

**Table 1:** Characteristics of included studies.

Author	Year	Country of Trial	Trial Name/Number	Types of Cancer	NOAC	LMWH	Number of NOAC Patients	Number of LMWH Patients	Primary Outcome
Ageno	2019	International	APEX	Genitourinary, gastrointestinal, skin, respiratory	Betrixaban	Enoxaparin	499	460	Deep vein thrombosis, pulmonary embolism
Agnelli	2015	International	AMPLIFY	Prostate, breast, colon, bladder, lung	Apixaban	Enoxaparin	88	81	Symptomatic venous thromboembolism or venous thromboembolism related death
Guntupalli	2018	USA	NCT02366871	Gynaecologic cancers (uterine, ovarian, cervical and vulvar)	Apixaban	Enoxaparin	204	196	Major bleeding and clinically relevant non major bleeding
Kim	2022	Europe	PRIORITY	Advanced upper gastrointestinal, hepatobiliary or pancreatic cancer	Rivaroxaban or apixaban	Dalteparin	44	46	Major bleeding and clinically relevant non major bleeding
McBane	2019	USA	ADAM-VTE	Solid tumour, haematologic	Apixaban	Dalteparin	145	142	Major bleeding and clinically relevant non major bleeding
Mokadem	2021	Egypt	NCT04462003	Colon, bladder, prostate, liver, ovary, uterus, breast	Apixaban	Enoxaparin	50	50	Major bleeding
Planquette	2022	France	CASTA DIVA	Colorectal, lung, breast, myeloma/lymphoma, prostate, pancreas or hepatobiliary/liver, kidney or bladder, uterus or ovary, upper gastrointestinal, head and neck, primary brain tumour	Rivaroxaban	Dalteparin	74	84	Recurrent venous thromboembolism
Raskob	2018	International	Hokusai	Cancers other than squamous cell carcinoma and basal cell carcinoma	Edoxaban	Dalteparin	522	524	Recurrent venous thromboembolism, major bleeding
Wang	2019	China	NCT03282643	Lung, breast, oesophageal/gastroesophageal, hepatocellular carcinoma, colorectal malignant tumour	Rivaroxaban	Nadroparin	51	23	Venous thromboembolism
Young	2018	International	Select-D	Solid, haematologic	Rivaroxaban	Dalteparin	203	203	Venous thromboembolism recurrence
Agnelli	2020	International	CARAVAGGIO	Patients with confirmed cancer other than basal-cell or squamous-cell carcinoma of the skin, primary brain tumour, known intracerebral metastases, or acute leukaemia	Apixaban	Dalteparin	576	579	Recurrent venous thromboembolism

## Data Availability

Data used for analysis can be made available upon reasonable request.

## Supplementary Materials

The following supporting information can be downloaded at: https://www.mdpi.com/article/10.3390/cancers15245887/s1, Supplementary Figure S1: Risk of bias assessment of included studies; Supplementary Data S1: EMBASE Search Strategy; Supplementary Data S2: Reconstructed Kaplan–Meier Curves; Supplementary Data S3: Forest plots of other meta-analysed outcomes.
