# Supplementary material for: Non-Vitamin K Antagonist Oral Anticoagulants versus Low Molecular Weight Heparin for Cancer-Related Venous Thromboembolic Events: Individual Patient Data Meta-Analysis
Source: Cancers (Basel). 2023 Dec 18;15(24):5887. doi: 10.3390/cancers15245887 (PMC10741613; doi:10.3390/cancers15245887)

Supplementary Figure S1: Risk of bias assessment of included studies

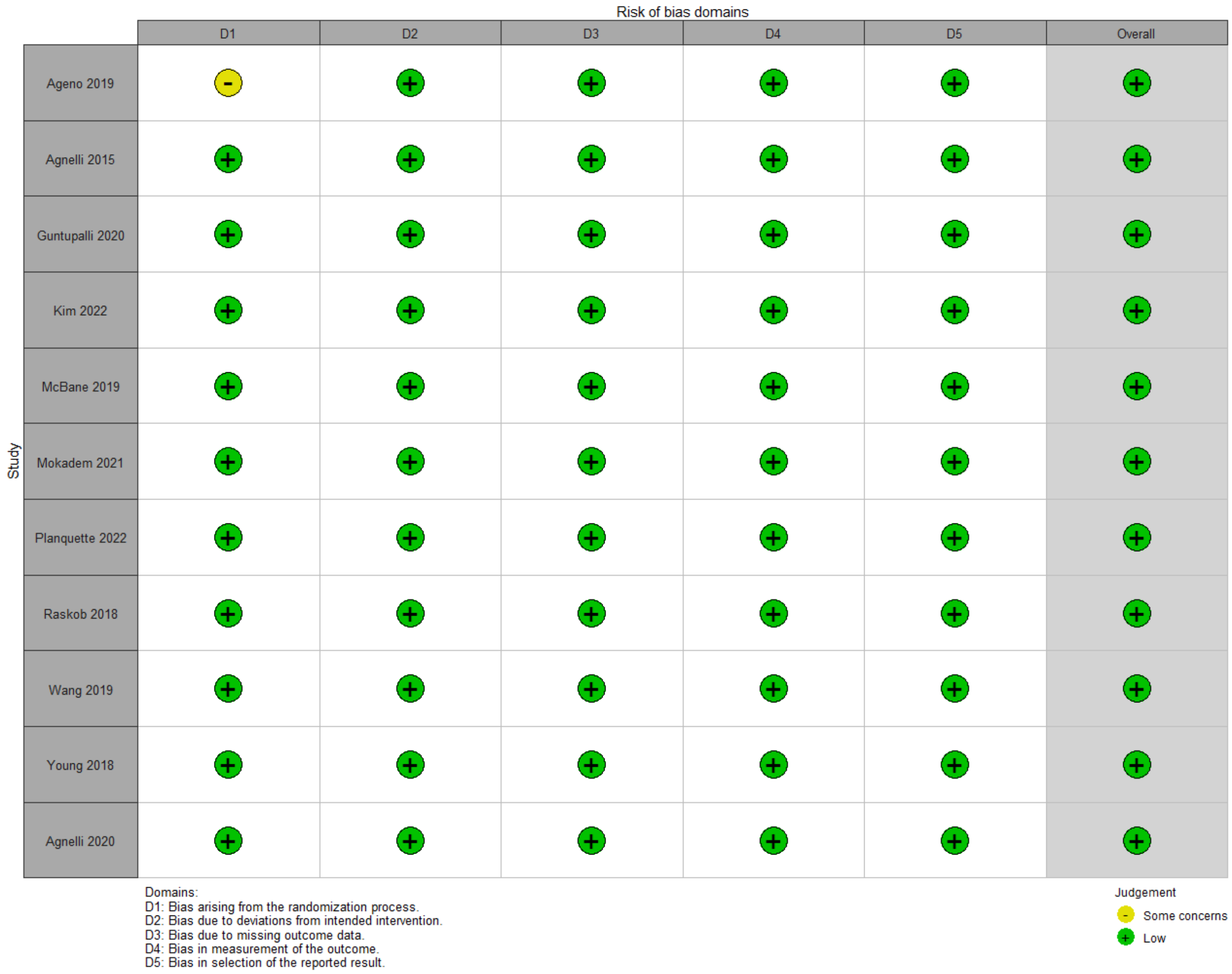

Supplementary Data S1: EMBASE Search Strategy

**1**	‘neoplasm':ti,ab OR 'cancer*':ti,ab OR 'tumo*':ti,ab OR 'malign*':ti,ab or "neoplasm"/exp
**2**	((vein* or venous* or deep-vein* or deep-veno*) NEAR/3 (occlu* or thrombo* or block* or stasis*)):ti,ab
**3**	"venous thromboembolism"/exp or "vein thrombosis"/exp or "lung embolism"/exp or "occlusive cerebrovascular disease "/exp or "budd chiari syndrome"/exp or "postthrombosis syndrome"/exp or "vein occlusion"/exp or "phlegmasia alba dolens"/exp or "Lemierre syndrome"/exp
**4**	("pulmonary embolism" or "Phlebothrombosis" or "budd-chiari syndrome" or "hepatic venous outflow obstruction" or "chiaris syndrome " or "chiari syndrome" or "postthrombotic syndrome" or "Thrombophlebit*" or "phlegmasia alba dolens" or "lemierre disease" or "lemierre syndrome" or "lemierres disease" or "lemierres syndrome" or "postanginal sepsis " or "paget-schroetter syndrome" or "paget schroetter" or "thromboembolism"):ti,ab
**5**	"blood clotting inhibitor"/exp OR 'new oral anticoagulant'/exp OR 'novel oral anticoagulant'/exp OR 'blood clotting factor 10a inhibitor'/exp OR ('apixaban*' OR 'betrixaban*' OR 'edoxaban*' OR 'rivaroxaban*' OR 'dabigatran*' or "Xarelto" or "BAY 59-7939" or "BMS 562247" or "BMS-562247" or "Xa inhibitor" or "Melagatran" or "Ximelagatran" or "Exanta" or "H 376 95" or "H 376-95" or "DU-176b" or "xi-melagatran" or "BMS562247" or "asundexian"):ti,ab
**6**	'low molecular weight heparin'/exp OR ('lmwh' OR 'enoxaparin' OR 'nadroparin' OR 'tinzaparin' OR 'dalteparin'):ti,ab

1 AND (2 OR 3 OR 4) AND 5 AND 6, limit to RCTs

Supplementary Data S2: Reconstructed Kaplan-Meier Curves

VTE recurrence – Raskob 2018 original

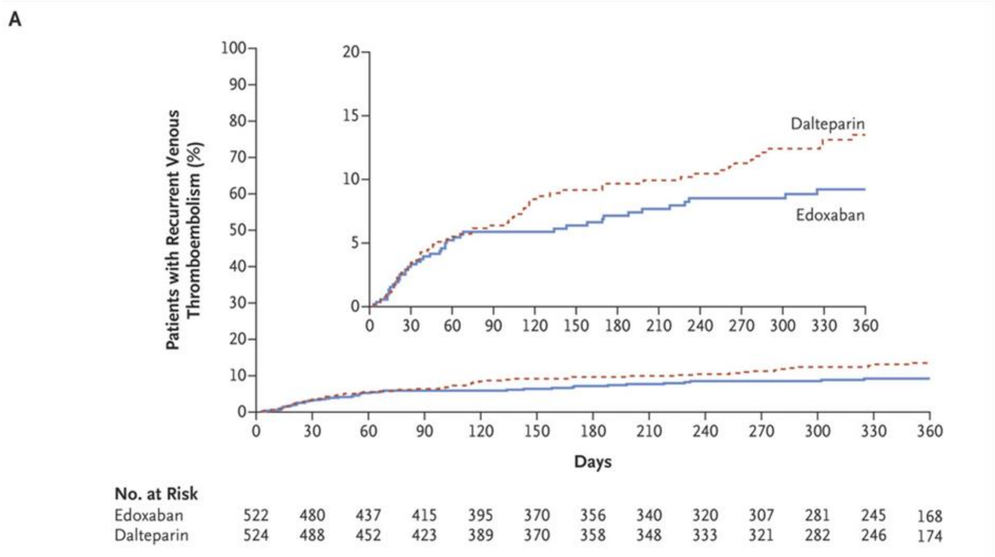

VTE recurrence – Raskob 2018 reconstructed

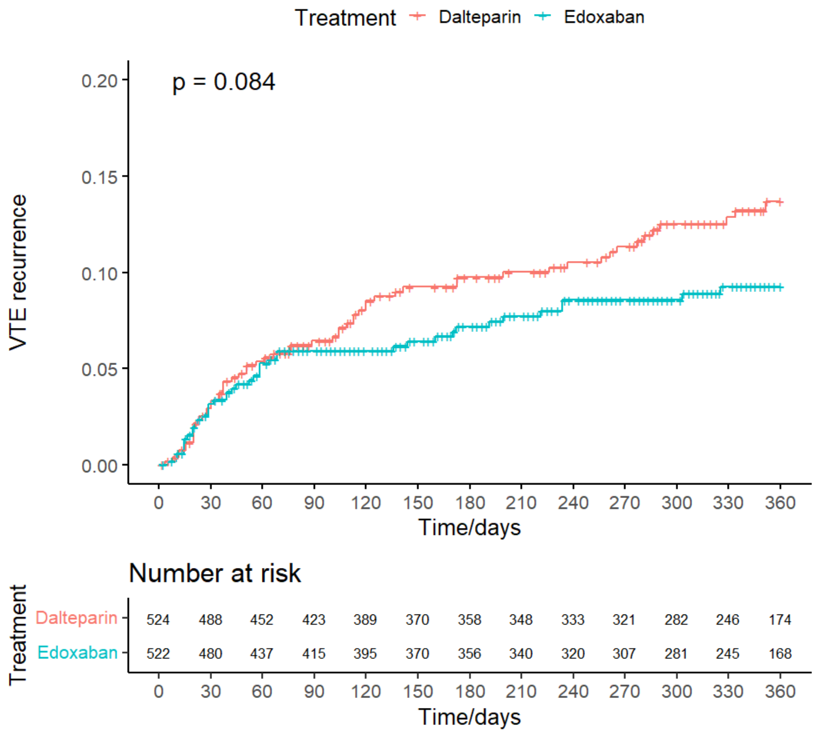

VTE recurrence – Agnelli 2020 original

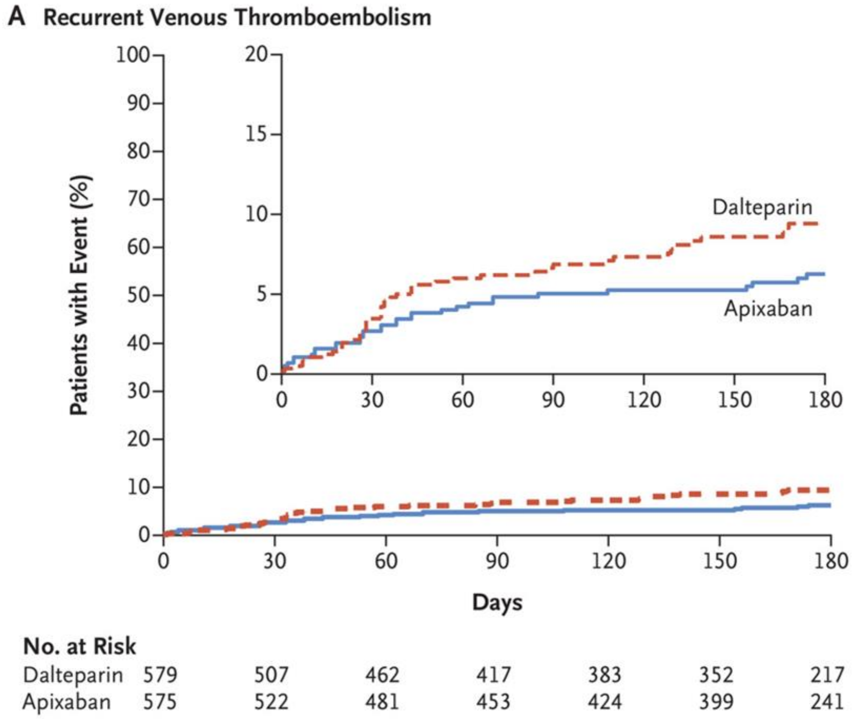

VTE recurrence – Agnelli 2020 reconstructed

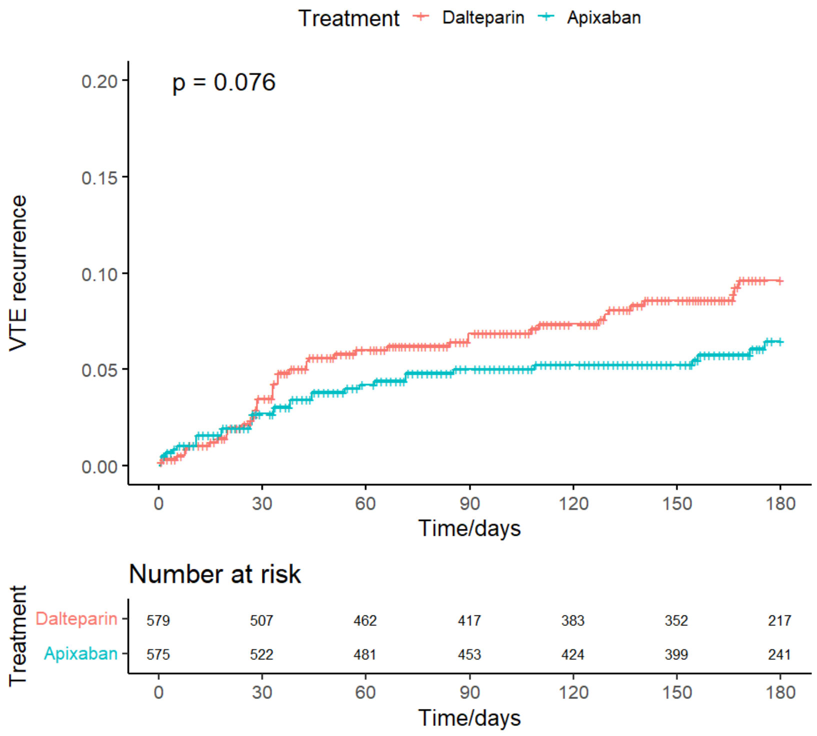

VTE recurrence – Young 2018 original

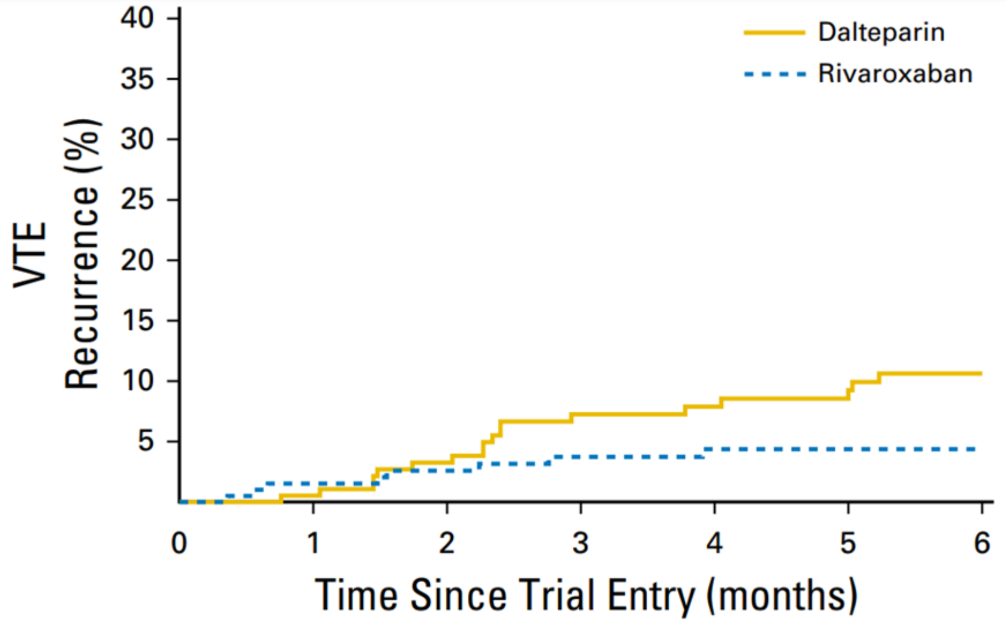

No. at risk:

Dalteparin	203	171	139	115
Rivaroxaban	203	174	149	134

VTE recurrence – Young 2018 reconstructed

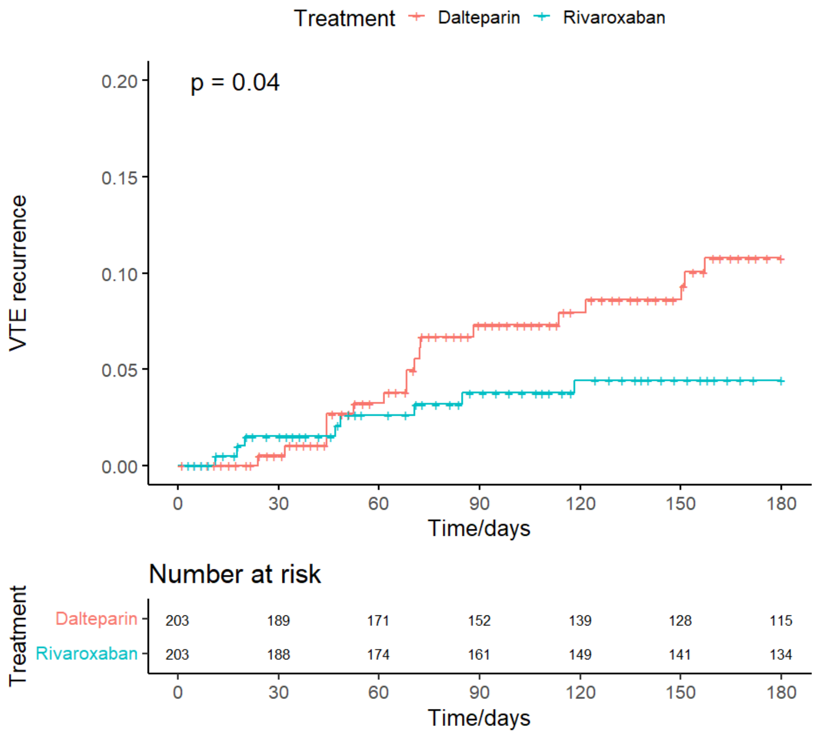

Major bleeding – Agnelli 2020 original

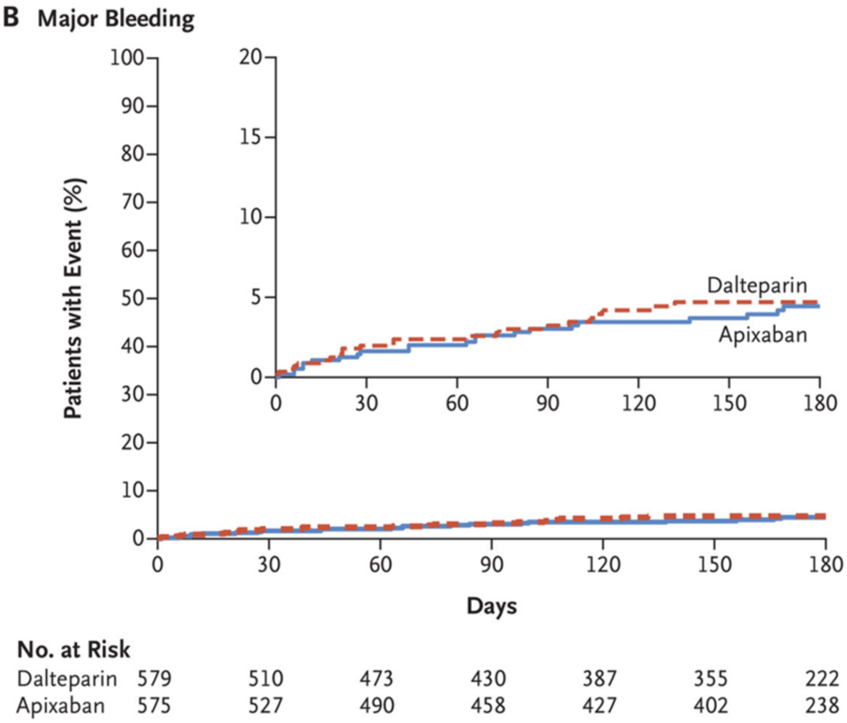

Major bleeding – Agnelli 2020 reconstructed

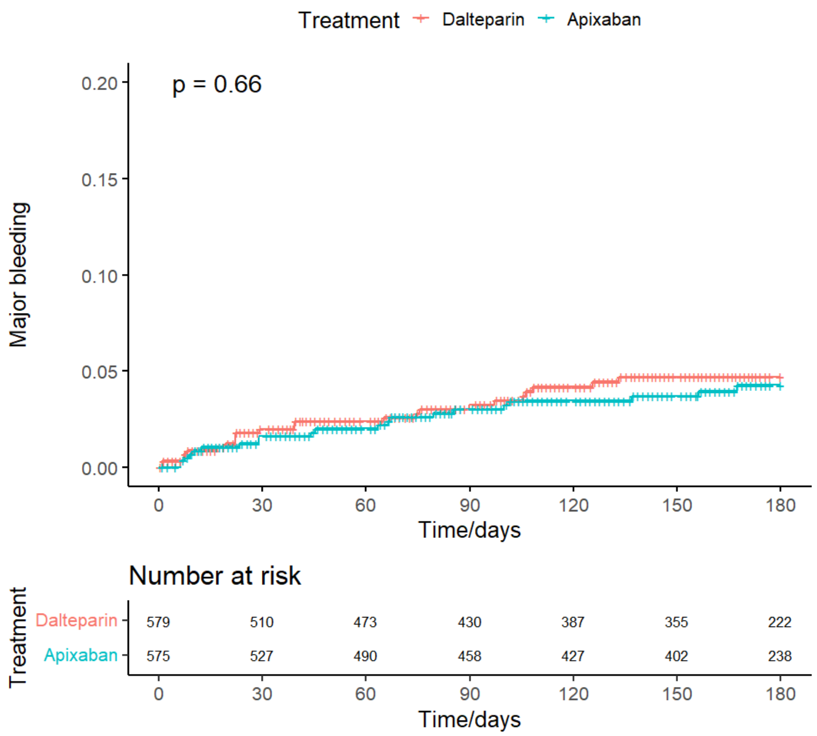

Major bleeding – Raskob 2018 original

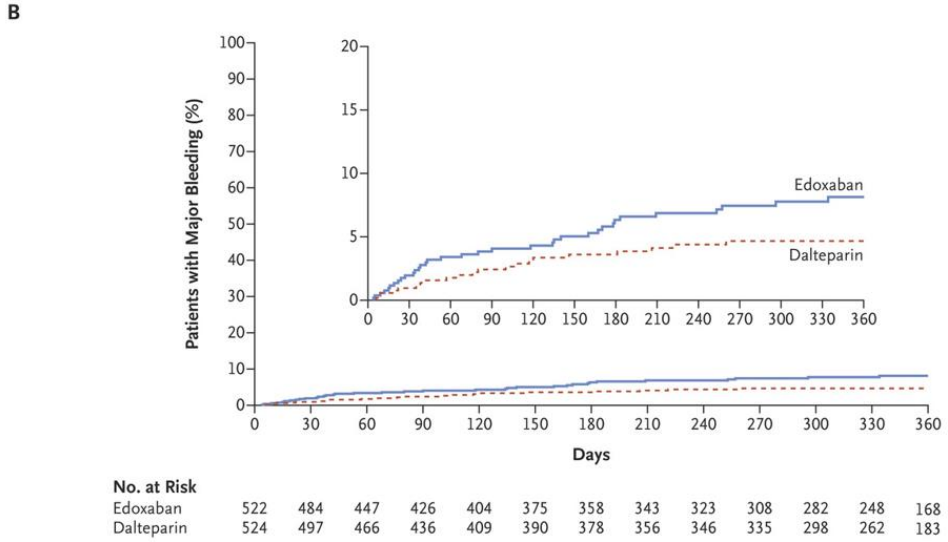

Major bleeding – Raskob 2018 reconstructed

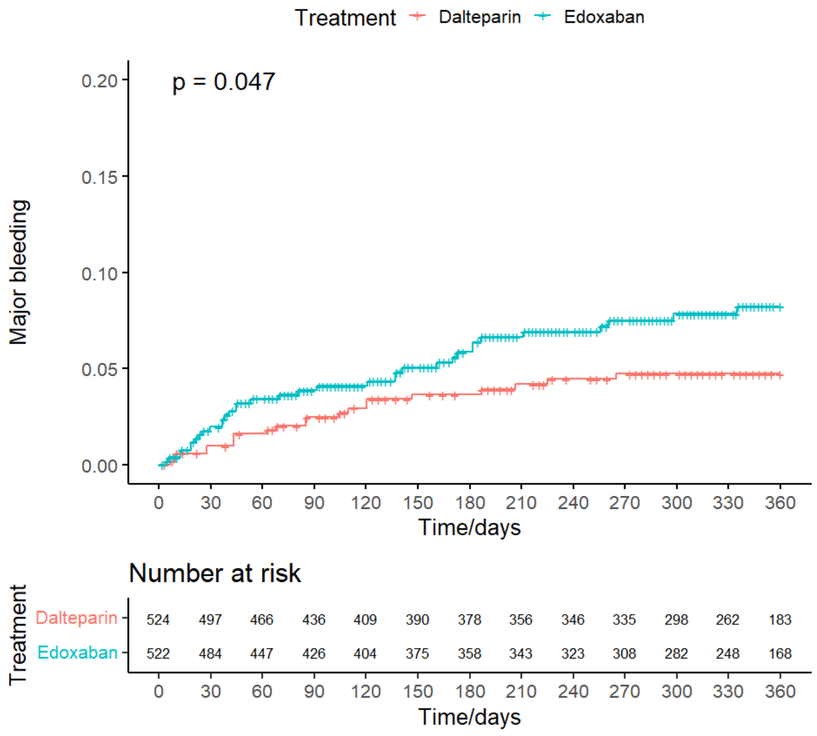

Supplementary Data 3: Forest plots of other meta-analysed outcomes

Forest plot of death due to pulmonary embolism

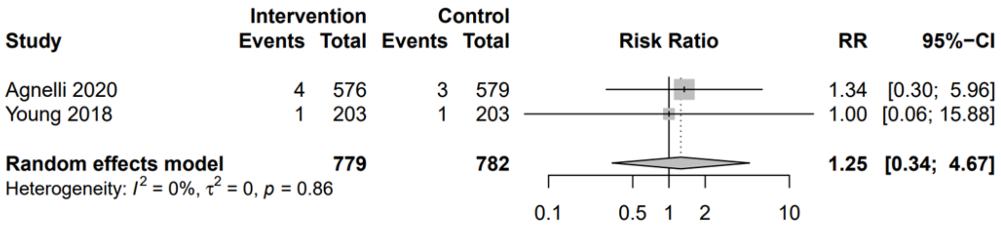

Forest plot of death due to bleeding

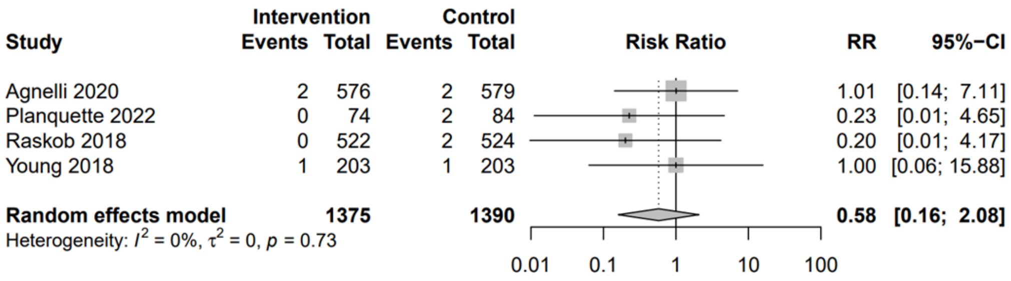

Forest plot of death due to venous thromboembolism

Study	Intervention Events	Intervention Total	Control Events	Control Total	Risk Ratio	RR	95%−CI
Ageno 2019	2	492	3	454		0.62	[0.10; 3.66]
Agnelli 2020	4	576	4	579		1.01	[0.25; 4.00]
Planquette 2022	0	74	0	84			
Raskob 2018	6	522	4	524		1.51	[0.43; 5.30]
**Random effects model**		**1664**		**1641**		**1.08**	**[0.47; 2.46]**

Heterogeneity: $I^2 = 0\%$, $\tau^2 = 0$, $p = 0.72$

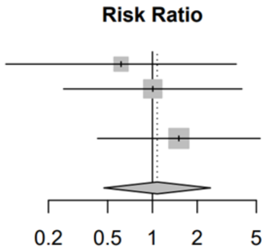

Forest plot of death outcomes

Study	Intervention Events	Intervention Total	Control Events	Control Total	Risk Ratio	RR	95%−CI
McBane 2019	23	145	15	142		1.50	[0.82; 2.76]
Agnelli 2020	135	576	153	579		0.89	[0.73; 1.08]
Planquette 2022	19	74	20	84		1.08	[0.63; 1.86]
Raskob 2018	206	522	192	524		1.08	[0.92; 1.26]
Mokadem 2021	15	50	11	50		1.36	[0.70; 2.67]
Young 2018	28	203	28	203		1.00	[0.61; 1.63]
**Random effects model**		**1570**		**1582**		**1.03**	**[0.90; 1.18]**

Heterogeneity: $I^2 = 0\%$, $\tau^2 = 0.0044$, $p = 0.46$

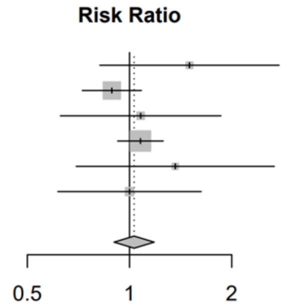

Forest plot of clinically relevant non-major bleeding outcomes

Study	Intervention Events	Intervention Total	Control Events	Control Total	Risk Ratio	RR	95%−CI
McBane 2019	9	145	7	142		1.26	[0.48; 3.29]
Agnelli 2015	9	87	14	80		0.59	[0.27; 1.29]
Ageno 2019	10	492	9	452		1.02	[0.42; 2.49]
Agnelli 2020	52	576	35	579		1.49	[0.99; 2.26]
Planquette 2022	8	74	5	84		1.82	[0.62; 5.31]
Raskob 2018	76	522	58	524		1.32	[0.96; 1.81]
Guntupalli 2020	12	204	19	196		0.61	[0.30; 1.22]
Mokadem 2021	2	50	3	50		0.67	[0.12; 3.82]
Kim 2022	8	44	4	46		2.09	[0.68; 6.45]
Young 2018	25	203	7	203		3.57	[1.58; 8.07]
**Random effects model**		**2397**		**2356**		**1.25**	**[0.89; 1.76]**

Heterogeneity: $I^2 = 46\%$, $\tau^2 = 0.1379$, $p = 0.05$

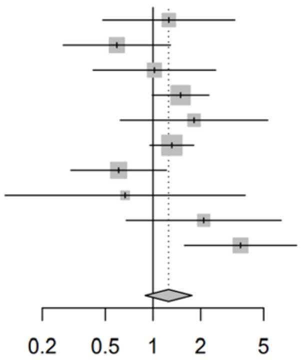

Forest plot of major or clinically relevant non-major bleeding outcomes

Study	Intervention Events	Intervention Total	Control Events	Control Total	Risk Ratio	RR	95%−CI
McBane 2019	9	145	9	142		0.98	[0.40; 2.40]
Ageno 2019	14	492	9	452		1.43	[0.62; 3.27]
Agnelli 2020	70	576	56	579		1.26	[0.90; 1.75]
Planquette 2022	9	74	8	84		1.28	[0.52; 3.14]
Raskob 2018	97	522	73	524		1.33	[1.01; 1.76]
Mokadem 2021	4	50	7	50		0.57	[0.18; 1.83]
Kim 2022	15	44	6	46		2.61	[1.12; 6.13]
**Random effects model**		**1903**		**1877**		**1.31**	**[1.08; 1.58]**

Heterogeneity: $I^2 = 0\%$, $\tau^2 < 0.0001$, $p = 0.54$

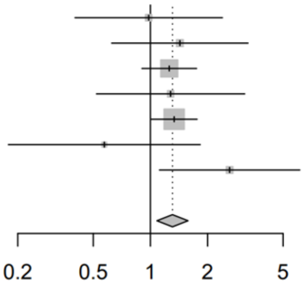

Forest plot of non-fatal pulmonary embolism outcomes

Study	Intervention Events	Intervention Total	Control Events	Control Total	RR	95%−CI
Ageno 2019	1	492	2	454	0.46	[0.04; 5.07]
Young 2018	3	203	8	203	0.38	[0.10; 1.39]
**Random effects model**		**695**		**657**	**0.39**	**[0.12; 1.24]**

Heterogeneity: $I^2 = 0\%$, $\tau^2 = 0$, $p = 0.88$

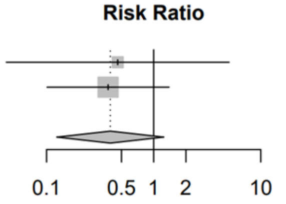

Forest plot of fatal pulmonary embolism outcomes

Study	Intervention Events	Intervention Total	Control Events	Control Total	RR	95%−CI
Agnelli 2020	4	576	3	579	1.34	[0.30; 5.96]
Young 2018	1	203	1	203	1.00	[0.06; 15.88]
**Random effects model**		**779**		**782**	**1.25**	**[0.34; 4.67]**

Heterogeneity: $I^2 = 0\%$, $\tau^2 = 0$, $p = 0.86$

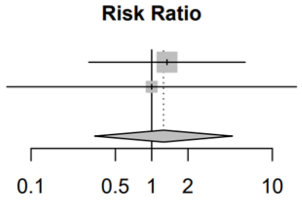

Forest plot of venous thromboembolism outcomes

Study	Intervention Events	Intervention Total	Control Events	Control Total	RR	95%−CI
Ageno 2019	28	492	28	454	0.92	[0.56; 1.53]
Guntupalli 2020	2	204	3	196	0.64	[0.11; 3.79]
Wang 2019	1	51	4	23	0.11	[0.01; 0.95]
Mokadem 2021	2	50	3	50	0.67	[0.12; 3.82]
**Random effects model**		**797**		**723**	**0.71**	**[0.37; 1.36]**

Heterogeneity: $I^2 = 18\%$, $\tau^2 = 0.0969$, $p = 0.30$

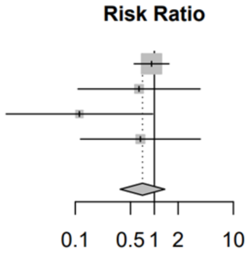

Forest plot of recurrent pulmonary embolism outcomes

Study	Intervention Events	Intervention Total	Control Events	Control Total	RR	95%−CI
McBane 2019	0	145	1	142	0.33	[0.01; 7.95]
Agnelli 2020	19	576	32	579	0.60	[0.34; 1.04]
Planquette 2022	1	74	2	84	0.57	[0.05; 6.13]
Raskob 2018	27	522	28	524	0.97	[0.58; 1.62]
Young 2018	4	203	9	203	0.44	[0.14; 1.42]
**Random effects model**		**1520**		**1532**	**0.71**	**[0.47; 1.06]**

Heterogeneity: $I^2 = 0\%$, $\tau^2 = 0.0272$, $p = 0.62$

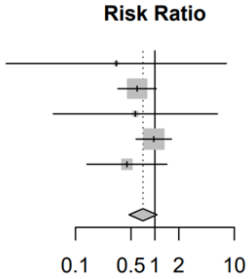

Supplement: Supplementary file 1 [file cancers-15-05887-s001.zip › cancers-2718476-supplementary.pdf]
